# Biomechanical Changes During Running on a Lower Body Positive Pressure Treadmill in Competitive Runners

**DOI:** 10.1111/sms.70063

**Published:** 2025-05-11

**Authors:** Dominik Fohrmann, Isabelle Winter, Alexander Simon, Dimitris Dalos, Thomas Gronwald, Tim Hoenig, Tim Rolvien, Karsten Hollander

**Affiliations:** ^1^ Institute of Interdisciplinary Exercise Science and Sports Medicine Faculty of Medicine, MSH, Medical School Hamburg Hamburg Germany; ^2^ Department of Osteology and Biomechanics University Medical Center Hamburg‐Eppendorf Hamburg Germany; ^3^ Department of Trauma and Orthopaedic Surgery University Medical Center Hamburg‐Eppendorf Hamburg Germany; ^4^ UKE Athleticum ‐ Center for Athletic Medicine University Medical Center Hamburg‐Eppendorf Hamburg Germany

**Keywords:** anti‐gravity treadmill, lyapunov exponent, running injuries, tibial shock

## Abstract

Lower body positive pressure treadmills (LBPPTs) offer precise body weight unloading for injury rehabilitation and performance training in runners. This study investigated biomechanical changes during running at varying body weight support (BWS) levels (0%–80%) in competitive runners, including sex‐specific responses. Twenty‐six runners (age: 33.6 ± 9.8 years; 15 female, 11 male) completed randomized 3‐min running bouts at 12 km/h across nine BWS levels. Spatiotemporal parameters, plantar force, peak tibial acceleration, and running stability were measured using pressure insoles and inertial sensors placed at the tibia and foot. Our results revealed significant reductions in step rate (*b* = −0.24 steps•min^−1^/%BWS, *p* < 0.001), normalized ground contact time (*b* = −0.001 1/%BWS, *p* < 0.001), maximum plantar force (*b* = −0.010 BW/%BWS, *p* < 0.001), and peak tibial acceleration (*b* = −0.03 g/%BWS, *p* < 0.001) with increased BWS. Swing time increased (*b* = 1.50 ms/%BWS, *p* < 0.001), while stance time decreased (*b* = −0.41 ms/%BWS, *p* < 0.001). Running stability showed marginal changes (foot: *b* = −0.001 1/%BWS, *p* = 0.017; tibia: *b* = 0.001 1/%BWS, *p* = 0.009). Sex differences were observed in step rate (*b* = −6.79 steps•min^−1^, *p* = 0.045) and maximum plantar force (*b* = −0.128 BW, *p* = 0.034), but there were no significant sex × BWS interaction effects for any of the investigated parameters. Findings from this study highlight the effectiveness of LBPPTs for reducing musculoskeletal loading while revealing associated gait changes. Athletes, therapists, and coaches should consider individual biomechanical responses to optimize rehabilitation and performance strategies. Future research should explore long‐term adaptations and injury prevention outcomes.

## 1 Introduction

Running is one of the primal human movements. It is one of the most popular recreational and competitive sports due to its high accessibility. Still, there is a high risk of injury. Incidence of running‐related injuries (RRIs) ranges from 2.5 times per 1000 h running exposure in experienced runners to 33 times per 1000 h running exposure in novice runners [1]. The overall injury rates are similar for sexes with 20.8 and 20.4 per 100 female and male runners, respectively [2]. Most running‐related injuries are caused by repetitive tissue loading (i.e., *overuse injuries*). For prevention and rehabilitation, optimal progressive load management is essential to allow for tissue adaptation and support physiological healing response in the return to running‐specific movements or sports [3]. This is particularly important, since the history of RRIs is a significant risk factor and predictor of future injuries [4].

One method that provides granular control of load during rehabilitation is the use of a lower body positive pressure treadmill (LBPPT). These treadmills generate upward lifting forces on the athlete by increasing air pressure in a chamber that surrounds both the athlete's lower body up to the waist and the treadmill running surface. LBPPTs allow for the selection of a desired unloading of up to 80% of body weight (BW), that is, 20% remaining BW. Compared to the alternatives such as underwater treadmills or harness systems, LBPPTs provide a more natural running environment and better control over the level of progressive reloading. This allows for an early return to running specific movements. Consequently, LBPPTs have been used in rehabilitation following bone stress injuries [5, 6], Achilles tendon ruptures [7], and severe knee injuries [8].

Running on an LBPPT is associated with certain biomechanical changes. Step rate and stance time decreased [9, 10, 11, 12], while stride length [13, 14] and flight time or swing time [10, 15] increased with unloading. Yet, these changes reached statistical significance only at increments of 20% or higher [10]. Peak tibial acceleration (PTA), on the other hand, was unaltered by unloading up to 40% [9]. Additionally, ground reaction forces, joint forces, and plantar forces have consistently been shown to decrease with unloading [11, 16, 17, 18, 19, 20].

In recent years, nonlinear analyses of running gait have gained research interest [21]. Running stability is one such parameter and quantifies the runner's ability to compensate for minor perturbations. These can be introduced through varying running surfaces [22], different footwear [23], or fatigue [24]. It can be measured from various sources of kinematic data. Physiological demands are reduced when running with increased body weight support (BWS) [25, 26]. Theoretically, this might improve running stability by freeing up neuromuscular resources. In practice, to our best knowledge, no study has investigated changes in running stability with varying BWS yet. Previous studies investigating changes in muscle activity of the lower limbs when running with changing BWS mainly showed reduced levels [15, 27]. This suggests that muscular efforts are altered, but it remains unclear how reduced BW on an LBPPT affects running stability. Also, even though multiple previous studies have shown sex‐specific differences in biomechanics [28, 29] and running injury profile and risk [2, 30], most studies investigating biomechanical changes on LBPPTs do not include both sexes or, if so, do not analyze the data separately [9, 12, 17].

To improve the current understanding of the effects of unloading when using an LBPPT on running biomechanics, the aim of the present study was to comprehensively investigate changes in kinematics, kinetics, and running stability during running at different levels of BWS. We hypothesized that running stability was increased and that PTA, step rate, maximum plantar force, and normalized ground contact time (nGCT) were decreased with increased BWS. We further explored whether male and female runners exhibited different responses to BWS in the investigated biomechanical parameters.

## 2 Methods

Reporting of this cross‐sectional study was guided by the “Strengthening the Reporting of Observational Studies in Epidemiology” (STROBE) statement [31]. The study was approved by the ethics board of the Medical School Hamburg (MSH‐2022/184) and followed the principles of the Declaration of Helsinki in its latest version.

### 2.1 Participants

Competitive long‐distance runners (tier two to tier four according to [32]) were recruited through local sports clubs, flyers in running stores, and word of mouth. The inclusion criteria were an official season's best time over the 10 km distance of at least 40 min for men and 45 min for women, age between 18 and 65 years, no lower limb injuries within 3 months prior to study participation, and regular participation in competitions or races. Simulation‐based power analyses based on previous findings [17, 33] with a significance level of 0.05 were performed using the simr package for mixed effects models [34]. The analyses indicated that a sample size of 22 would achieve a target power of 0.80 for the main effect BWS. However, due to the unknown effect sizes for running stability as well as potential dropouts, we aimed for a slightly higher sample size of *n* = 30. All participants gave informed written consent. Participants were not informed about the measurement outcomes or hypotheses.

### 2.2 Protocol

**FIGURE 1:**
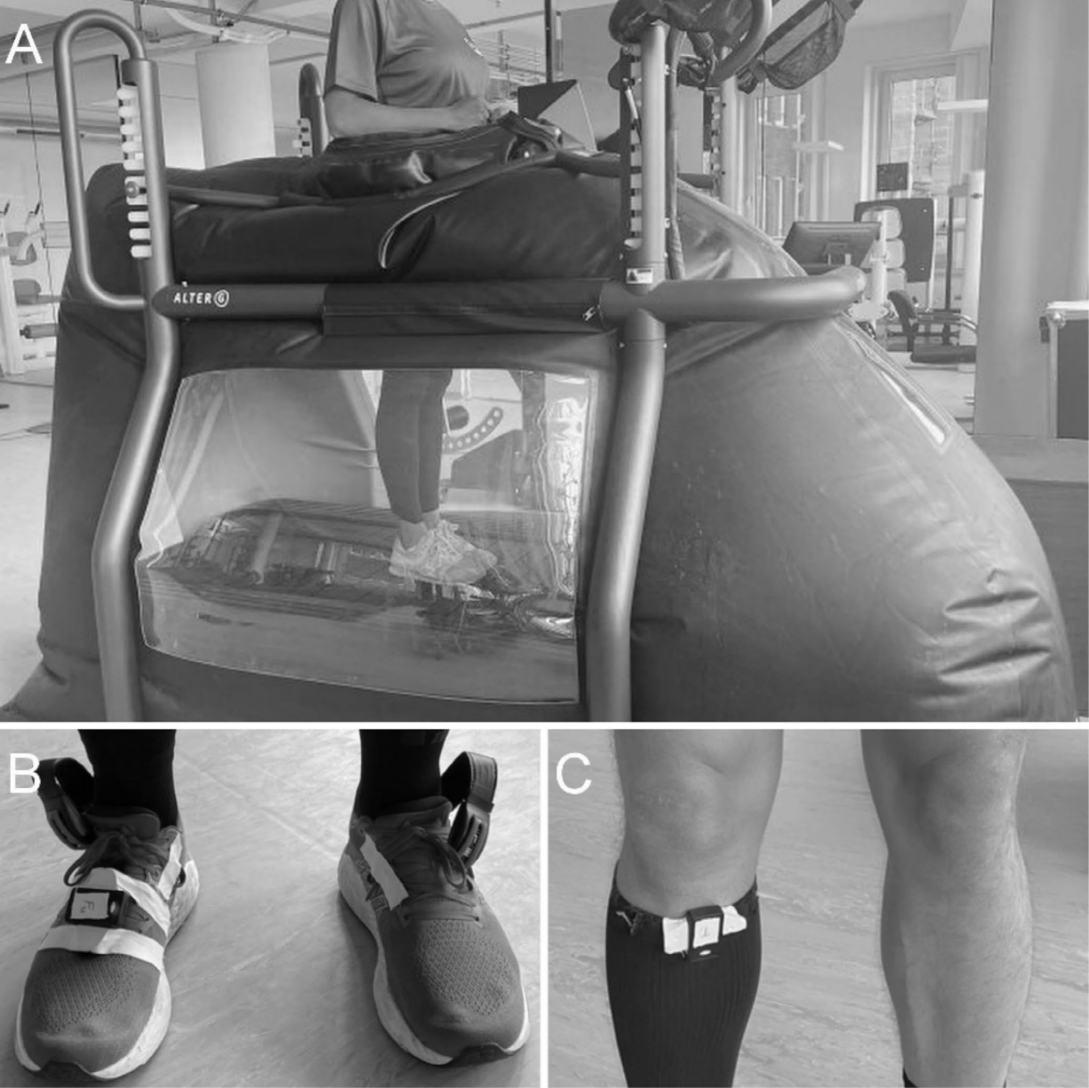
Instruments used in this study. (A) lower body positive pressure treadmill (LBPPT), fully inflated. The air chamber is attached to the bars around the athlete's waist where it is zipped to the neoprene skirt. (B) In‐shoe pressure measuring soles and foot inertia sensor. The soles corresponding data logger was attached to the lacing. The IMU was attached in the front of the lacing with push buttons. (C) Tibial inertia sensor. The IMU was attached to an elastic calf sleeve with push buttons and positioned to the medial proximal part of the tibia.

All measurements took place at the sports and performance laboratory of the Institute of Interdisciplinary Exercise Science and Sports Medicine, MSH Hamburg, during a single visit between January 2023 and March 2024. After personal instruction and the opportunity for questions, body height and weight were measured (see section “data collection”). Subsequently, participants were equipped with properly sized neoprene pants to accommodate the LBPPT chamber (Figure 1A). Then, participants were zipped into the air pressure chamber. The bars of the treadmill's support frame were aligned to the participant's iliac crest as recommended by De Heer et al. [35]. Calibration of the antigravity treadmill was performed according to the manufacturer's instructions.

The running protocol was conducted on an LBPPT (AlterG Pro 500, AlterG Inc., Fremont, CA, USA, Figure 1A). After a familiarization and warm‐up phase, the experimental phase followed. During familiarization, participants walked at 5 km/h for 1 min at each of five BWS levels (0%BWS, 20%BWS, 40%BWS, 60%BWS, and 80%BWS), totaling 5 min. The warm‐up followed the same sequence at 10 km/h, lasting another 5 min. After a 1‐min pause to initiate the measurement devices, the testing protocol commenced. The experimental phase involved nine consecutive 3‐min running bouts at a speed of 12 km/h, with BWS levels randomized between 0% and 80% in 10% increments (randomized using random.org). Thus, the entire protocol lasted 38 min. The running speed was intended to be submaximal for the intended population and is well within the range of previous studies [19, 36]. The individual protocols were created as XML files and uploaded to the treadmill via its USB interface.

Participants wore their own preferred running shoes during the measurements. Any orthopedic insoles were removed to allow for sufficient space for the pressure sensing insoles (see section “data collection”); except for one case where orthopedic insoles were necessary to prevent pain during running. Participants were not encouraged or instructed to change their running technique in any way.

### 2.3 Data Collection

Throughout the protocol, continuously recorded in‐shoe plantar pressure was recorded using two pressure‐sensing insoles with three sensor zones for the rearfoot, medial forefoot, and lateral forefoot (200 Hz, loadsol, novel GmbH, Munich, Germany, peak force ICC: 0.84–0.99 [37]). Before the start of the recording, a zero‐offset calibration was performed. Three‐dimensional acceleration and angular velocity were measured using two custom‐built inertial sensors at the foot and tibia (500 Hz, gyroscope: ±4000°/s, accelerometer: ±32 g, ICM 20601, TDK InvenSense, San Jose, CA, USA, ±2% sensitivity scale factor tolerance, ±60 mg zero‐g offset). The foot sensor was attached with a custom knob system through the shoelaces and further secured using adhesive tape (Figure 1B). The tibia sensor was attached using a knob system integrated into elastic calf sleeves (Figure 1C). Body weight and height were measured using a combined weight and height scale (655‐US, Seca GmbH & Co KG, Hamburg, Germany). Immediately after the runs, participants were asked about their rate of perceived exertion (RPE) for the running protocol overall using the modified CR10 Borg scale (0–10) [38].

### 2.4 Data Processing

Raw pressure and inertial sensor data were processed with custom Python scripts (version 3.10, python.org). Both continuous insole and inertial sensor data were initially cut into 3‐min trials by visually detecting the start of the experimental phase in the raw signals. Data from the pressure insoles was provided in total force, as well as the single forces of the three measurement zones. These forces represent the normal forces with respect to the foot contact area and should not be confused with true vertical ground reaction forces. From the total force, we determined the running gait events’ initial contact and terminal contact with a threshold of 50 N. Stride time, contact time, and swing time were calculated from the difference in milliseconds between consecutive ipsilateral initial contact events, initial and terminal contact events, and terminal and initial contact events, respectively. The nGCT was calculated as the ratio of contact time over stride time of the same stride cycle. Long‐term measurements during pilot testing showed that there can be a time drift between the internal clocks of the two soles. Thus, we could not determine the true step rate. Instead, we calculated the stride frequency for both sides separately, multiplied both values by two, and report the average of both sides as the step rate. Maximum plantar force (F_max_), expressed in BW units, was calculated as the peak value of the total force signal recorded between initial and terminal contact. This value was then normalized by dividing it by the force equivalent of BW, derived from the measured BW multiplied by the gravitational constant.

Since the inertial sensors were not time‐synchronized, we performed gait event detection separately for each sensor. For the tibia sensor, we initially applied a previously reported algorithm [39]. However, the method proved unreliable upon visual inspection, particularly in the higher BWS conditions. Consequently, we adopted another approach using the gyroscope signals as described by Falbriard et al. [40]. In brief, we identified the mid‐swing phase by detecting the maximum of the sagittal plane gyroscope signal to separate consecutive strides. The subsequent negative zero‐crossing of the signal marked the most anterior position of the foot in the late swing. We utilized the first maximum of the longitudinal acceleration signal after this zero‐crossing with a minimum height of 95% of the stride signal as both IC and PTA events. A systematic visual inspection showed a robust detection of the PTA over all BWS levels. While this procedure is not a validated approach for the correct IC event detection across varying foot strike types, it is a robust estimate and sufficiently accurate for stride separation needed for the calculation of running stability. From the foot signals, we identified the IC events from the minimum of the pitch‐gyroscope signal of the foot‐mounted sensor as described as k1 by Falbriard et al. [40].

Running stability was computed as the maximum short‐term finite‐time divergence exponent as described previously [41]. We used the three‐dimensional gyroscope signals of the last 100 consecutive strides of each BWS condition and resampled the signals to 10.000 samples. For the time‐delayed embedding, we chose a time delay of 11 time‐normalized samples for the foot and 12 samples for the tibia, resulting from the minimum of the average mutual information [42]. The subsequent global false nearest neighbor analysis yielded embedding dimensions of nine for the foot and six for the tibia sensor [43]. Utilizing Rosenstein's algorithm, we computed the logarithmic rate of divergence of initial nearest neighbors [44]. We fitted lines through the first 30 (foot) and 20 (tibia) samples of the resulting divergence curves after visual inspection. The slope of these lines yielded the largest short‐term finite time divergence exponent as the metric for running stability.

### 2.5 Statistical Analysis

Mean and standard deviation (SD) of spatiotemporal parameters (step rate, stance time, swing time, and nGCT), F_max_, and PTA were calculated for each condition. Data of the last 30 s of each 3‐min bout were analyzed to allow adjusting to the new BWS condition. Computation of running stability yielded a single value per participant per BWS condition for both foot and tibia sensors. To analyze the effect of BWS on the biomechanical outcomes and differences between male and female runners, we used linear mixed effects random intercept models (random intercepts for each participant). Significance testing was performed using Satterthwaite's method for degrees of freedom approximation using RStudio (version 2023.06.0, RStudio Team, Boston, MA, USA). Each model included the %BWS, sex (factorized as male = 1, female = 2), and the interaction as fixed effects. Marginal and conditional *R* ^2^ were computed as indicators of model fit using the MuMIn package (version 1.47.5). We report the standardized coefficients β of the fixed effects as an indicator of the effect size and the unstandardized estimates b for improved interpretability. Reported *p* values of the fixed effects refer to the unstandardized model fit. The level of significance was set at alpha = 0.05.

## 3 Results

### 3.1 Participant's Characteristics

A total of 35 participants were recruited, all of whom completed the entire protocol. Due to device measurement or connection errors of either the pressure insoles or inertial sensors, seven measurements were interrupted. Another two insole datasets showed disproportioned signal quality upon inspection. Hence, a total of 26 runners (15 female (57.7%), age: 33.6 ± 9.8 years, BMI: 21.6 ± 2.4 kg/m^2^) were included in the final analysis. The descriptive statistics of the sample in total and specified by sex are displayed in Table 1. RPE values had a total mean of 2.6 ± 0.9 with a maximum of 5.

**TABLE 1:** Sex‐specific demographics of the cohort.

	Male M ± SD (range)	Female M ± SD (range)	Total M ± SD (range)
Age (years)	37.2 ± 10.7 (22–58)	31.0 ± 8.5 (20–46)	33.6 ± 9.8 (20–58)
Height (cm)	178.0 ± 5.8 (164.0–185.0)	169.1 ± 7.2 (161.0–186.0)	172.8 ± 7.9 (161.0–186.0)
Weight (kg)	74.7 ± 8.9 (62.2–95.3)	58.2 ± 8.1 (46.7–75.8)	65.2 ± 11.7 (46.7–95.3)
BMI (kg·m^−2^)	23.5 ± 1.8 (21.4–28.5)	20.3 ± 1.9 (16.7–24.6)	21.6 ± 2.4 (16.7–28.5)
RPE (0–10)	2.9 ± 0.9 (2–5)	2.4 ± 0.7 (1–4)	2.6 ± 0.9 (1–5)
Number of Participants	11	15	26

Abbreviations: BMI: body mass index; RPE: rating of perceived exertion.

### 3.2 Spatiotemporal Parameters: Step Rate, Stance Time, Swing Time, and nGCT

**FIGURE 2:**
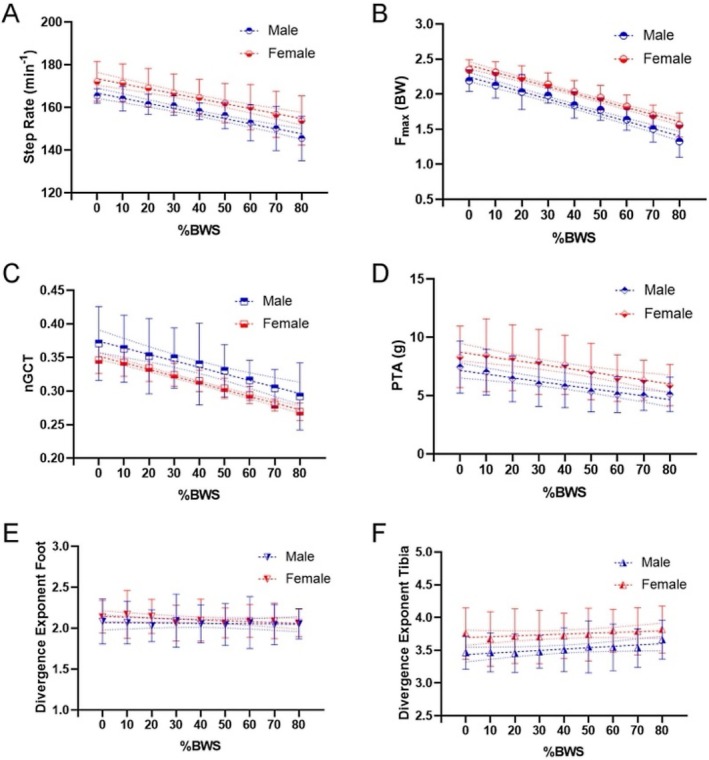
Linear regression results comparing male (*n* = 11) and female (*n* = 15) participants. Values are depicted as mean with standard deviation as error bars and 95% CI as dotted line. (A) Step rate to percentage of body weight support %BWS. (B) Maximum plantar force (F_max_) normalized with body weight gravitational force to percentage of body weight support %BWS. (C) Normalized ground contact time (nGCT) to percentage of body weight support %BWS. (D) Peak tibial acceleration (PTA) to percentage of body weight support %BWS. (E) Stability at the foot to percentage of body weight support %BWS. (F) Stability at the Tibia to percentage of body weight support %BWS.

**FIGURE 3:**
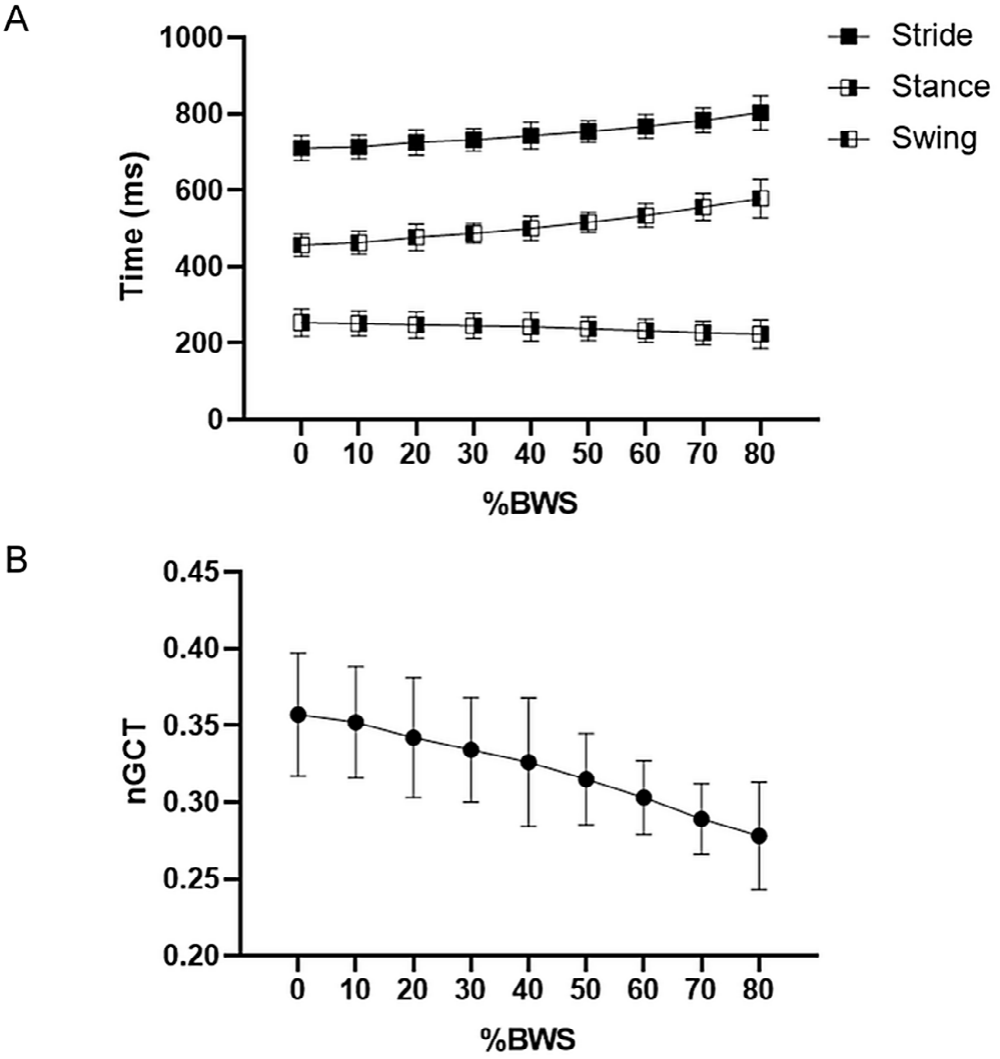
Data for (A) swing time, stance time, and stride time and (B) normalized ground contact time (nGCT) to percentage of body weight support (%BWS) of all participants. Values are depicted as means with standard deviations shown as error bars (*n* = 26).

Descriptive results for all investigated parameters over all BWS levels are presented in Table 3. All spatiotemporal parameters were significantly influenced by the level of BWS. In contrast, none of the spatiotemporal parameters showed a significant interaction effect of BWS and sex (step rate: *p*_BWS×Sex_ = 0.855, stance time: *p*_BWS×Sex_ = 0.081, swing time: *p*_BWS×Sex_ = 0.519, nGCT: *p*_BWS×Sex_ = 0.779). Step rate significantly decreased with increasing BWS (−0.24 ± 0.01 steps•min^−1^/%BWS, *β*_BWS_ = −0.56, 95% CI: [−0.61 to −0.50], *t*_BWS_ = −19.13, *p*_BWS_ < 0.001, Figure 2A). Furthermore, we found a significant difference between sexes in step rate (*β*_Sex_ = −0.63, 95% CI: [−1.20 to −0.07], *t*_Sex_ = −2.10, *p*_Sex_ = 0.045), indicating that female runners had a significantly lower step rate overall. The fixed effects explained 40% of the variance in step rate (*R* ^2^_marginal_ = 0.40), while the full model, including random participant effects, explained 89% (*R* ^2^_conditional_ = 0.89). Stance time significantly decreased with increasing BWS (−0.4 ± 0.0 ms/%BWS, *β*_BWS_ = −0.33, 95% CI: [−0.79 to 0.12], *t*_BWS_ = −18.86, *p*_BWS_ < 0.001, Figure 3A) with no significant sex difference (*β*_Sex_ = 0.79, 95% CI: [0.9–1.49], *t*_Sex_ = 2.03, *p*_Sex_ = 0.053). The fixed effects explained 22% of the variance in stance time (*R* ^2^_marginal_ = 0.22), with the full model explaining 97% (*R* ^2^_conditional_ = 0.97). Swing time significantly increased with increasing BWS (1.5 ± 0.1 ms/%BWS, *β*_BWS_ = 0.74, 95% CI: [0.67 to 0.82], *t*_BWS_ = 19.12, *p*_BWS_ < 0.001, Figure 3A) with no significant effect of sex (*p*_Sex_ = 0.986). The fixed effects explained 57% of the variance (*R* ^2^_marginal_ = 0.57), and the full model explained 80% (*R* ^2^_conditional_ = 0.80). Normalized ground contact time significantly decreased with increasing BWS (−0.001 ± 0.000 1/%BWS, *β*_BWS_ = −0.61, 95% CI: [−0.66 to −0.56], *t*_BWS_ = −23.42, *p*_BWS_ < 0.001, Figure 3B) with no significant effect of sex (*p*_Sex_ = 0.086, Figure 2C). The fixed effects accounted for 42% of the variance (*R* ^2^_marginal_ = 0.42), with the full model explaining 91% (*R* ^2^_conditional_ = 0.91).

### 3.3 Maximum Force

Maximum plantar force (i.e., normal force) significantly decreased with increasing BWS (−0.010 ± 0.000 BW/%BWS, *β*_BWS_ = −0.81, 95% CI: [−0.86 to −0.76], *t*_BWS_ = −30.14, *p*_BWS_ < 0.001, Figure 2B). Female runners exhibited a lower maximum force overall compared to male runners (*β*_Sex_ = −0.48, 95% CI: [−0.81 to −0.14], *t*_Sex_ = −2.22, *p*_Sex_ = 0.034). There was no significant interaction of BWS and sex (*p*_BWS×Sex_ = 0.243). The fixed effects explained 73% of the variance in maximum force (*R* ^2^_marginal_ = 0.73), and the full model explained 90% (*R* ^2^_conditional_ = 0.90).

### 3.4 Peak Tibial Acceleration

Peak tibial acceleration significantly decreased with increasing BWS (−0.03 ± 0.00 g/%BWS, *β*_BWS_ = −0.37, 95% CI: [−0.43 to −0.30], *t*_BWS_ = −11.09, *p*_BWS_ < 0.001, Figure 2D). There were no significant differences between male and female runners (*p*_Sex_ = 0.069) or interaction effects of BWS and sex (*p*_BWS×Sex_ = 0.527). The fixed effects accounted for 20% of the variance in PTA (*R* ^2^_marginal_ = 0.20) and the full model explained 86% (*R* ^2^_conditional_ = 0.86).

### 3.5 Running Stability

The effects of BWS on running stability were significant but minimal for both the foot and tibia (foot: −0.001 ± 0.000 1/%BWS, *β*_BWS_ = −0.12, 95% CI: [−0.22 to −0.02], *t*_BWS_ = −2.40, *p*_BWS_ = 0.017, Figure 2E; tibia: 0.001 ± 0.001 1/%BWS, *β*_BWS_ = 0.10, 95% CI: [0.02–0.17], *t*_BWS_ = −2.65, *p*_BWS_ = 0.009, Figure 2F). There were no significant differences between sexes in running stability overall (foot: *p*_Sex_ = 0.382; tibia: p_Sex_ = 0.061) or in interaction with BWS (foot: *p*_BWS×Sex_ = 0.241; tibia: *p*_BWS×Sex_ = 0.363). The fixed effects explained only 2% and 11% of the variance in running stability (foot: *R* ^2^_marginal_ = 0.02, tibia: *R* ^2^_marginal_ = 0.11), while the full model explained 13% and 83% for foot and tibia, respectively (foot: *R* ^2^_conditional_ = 0.13; tibia: *R* ^2^_conditional_ = 0.83). See Table 2 for detailed results of the linear mixed‐effects analysis for all parameters.

**TABLE 2:** Results for the fixed effects body weight support (BWS), sex, and the interaction of both (BWS × sex) from the linear mixed effects analysis. Presented estimates are the unstandardized coefficients. See the text for standardized estimates.

Fixed effects		Estimate [95% CI]		SE	*t* stat.	df	*p*
Step rate	Intercept	173.49	[169.38 to 177.59]	2.10	82.46	26.84	< 0.001
	BWS	−0.24	[−0.26 to −0.21]	0.01	−19.13	206.00	< 0.001
	Sex	−6.79	[−13.10 to −0.48]	3.23	−2.10	26.84	0.045
	BWS × Sex	0.00	[−0.04 to 0.03]	0.02	−0.18	206.00	0.855
Stance time	Intercept	245.43	[229.36 to 261.51]	8.2	29.84	24.6	< 0.001
	BWS	−0.41	[−0.46 to −0.37]	0.0	−18.86	206.0	< 0.001
	Sex	25.73	[1.02–50.44]	12.6	2.03	24.6	0.053
	BWS × Sex	0.06	[−0.01 to 0.12]	0.0	1.76	206.0	0.081
Swing time	Intercept	445.85	[431.16 to 460.53]	7.6	59.03	34.9	< 0.001
	BWS	1.50	[1.35 to 1.66]	0.1	19.12	206.0	< 0.001
	Sex	0.20	[−22.38 to 22.77]	11.6	0.02	34.9	0.986
	BWS × Sex	0.08	[−0.16 to 0.31]	0.1	0.65	206.0	0.519
nGCT	Intercept	0.35	[0.34 to 0.37]	0.01	42.63	26.2	< 0.001
	BWS	−0.001	[−0.001 to −0.001]	0.00	−23.42	206.0	< 0.001
	Sex	0.02	[0.00 to 0.05]	0.01	1.78	26.2	0.086
	BWS × Sex	0.00	[0.00 to 0.00]	0.00	0.28	206.0	0.779
F_max_	Intercept	2.383	[2.310 to 2.456]	0.04	63.45	31.2	< 0.001
	BWS	−0.010	[−0.011 to −0.009]	0.00	−30.14	206.00	< 0.001
	Sex	−0.128	[−0.241 to −0.016]	0.06	−2.22	31.2	0.034
	BWS × Sex	−0.001	[−0.002 to 0.000]	0.00	−1.17	206.0	0.243
PTA	Intercept	8.73	[7.68 to 9.78]	0.54	16.19	26.7	< 0.001
	BWS	−0.03	[−0.04 to −0.03]	0.00	−11.09	206.0	< 0.001
	Sex	−1.57	[−3.19 to 0.05]	0.83	−1.89	26.7	0.069
	BWS × Sex	0.00	[−0.01 to 0.01]	0.00	0.63	206.0	0.527
Running stability (Foot)	Intercept	2.143	[2.038 to 2.249]	0.054	39.68	30.3	< 0.001
	BWS	−0.001	[−0.002 to 0.000]	0.000	−2.40	206.0	0.017
	Sex	−0.074	[−0.235 to 0.088]	0.083	−0.89	30.3	0.382
	BWS × Sex	0.001	[−0.001 to 0.002]	0.001	1.18	206.0	0.241
Running stability (Tibia)	Intercept	3.692	[3.525 to 3.859]	0.086	43.08	27.0	< 0.001
	BWS	0.001	[0.000 to 0.002]	0.001	2.65	206.0	0.009
	Sex	−0.257	[−0.514 to 0.000]	0.132	−1.95	27.0	0.061
	BWS × Sex	0.001	[−0.001 to 0.002]	0.001	0.91	206.0	0.363

Abbreviations: BWS, body weight support; df, degrees of freedom; F_max_, body‐weight‐normalized maximum plantar force; nGCT, normalized ground contact time; PTA, peak tibial acceleration; SE, standard error.

**TABLE 3:** Overview of all variables over all BWS levels. Values are M ± SD and [95% CI].

BWS (%)	PTA (g)	Stance time (ms)	Swing time (ms)	Step rate (steps•min^−1^)	nGCT ()	F_max_ (BW)	Running stability foot (div. exponent)	Running stability tibia (div. exponent)
0	7.94 ± 2.47 [3.80 to 15.10]	254.60 ± 35.82 [220.19 to 340.76]	455.58 ± 28.83 [381.58 to 498.59]	169.31 ± 8.10 [158.20 to 182.10]	0.36 ± 0.04 [0.32 to 0.41]	2.27 ± 0.14 [2.04 to2.54]	2.11 ± 0.23 [1.76 to 2.41]	3.63 ± 0.37 [3.16 to 4.11]
10	7.85 ± 2.74 [4.44 to 11.65]	251.72 ± 33.47 [221.65 to 355.13]	462.54 ± 30.03 [425.87 to 527.91]	168.38 ± 8.43 [155.57 to 185.33]	0.35 ± 0.04 [0.28 to 0.41]	2.22 ± 0.17 [1.72 to 2.45]	2.12 ± 0.28 [1.71 to 2.76]	3.58 ± 0.38 [3.10 to 4.47]
20	7.48 ± 2.60 [3.75 to 13.31]	248.50 ± 35.20 [203.42 to 301.32]	476.46 ± 34.31 [433.17 to 515.89]	165.90 ± 8.42 [154.25 to 183.59]	0.34 ± 0.04 [0.29 to 0.48]	2.13 ± 0.21 [1.72 to 2.25]	2.09 ± 0.21 [1.83 to 2.43]	3.61 ± 0.39 [3.11 to 4.23]
30	7.08 ± 2.60 [3.74 to 14.93]	246.02 ± 33.16 [207.67 to 354.21]	486.31 ± 25.46 [437.97 to 530.51]	164.20 ± 7.85 [153.17 to 182.34]	0.34 ± 0.03 [0.30 to 0.38]	2.06 ± 0.15 [1.89 to 2.32]	2.07 ± 0.26 [1.75 to 2.88]	3.61 ± 0.36 [2.99 to 4.13]
40	6.85 ± 2.42 [3.58 to 12.05]	243.05 ± 38.87 [206.99 to 312.43]	499.83 ± 32.15 [390.14 to 542.93]	161.88 ± 7.57 [155.68 to 175.01]	0.33 ± 0.04 [0.28 to 0.43]	1.94 ± 0.18 [1.75 to 2.25]	2.08 ± 0.24 [1.76 to 2.54]	3.63 ± 0.35 [3.18 to 4.16]
50	6.33 ± 2.29 [3.27 to 10.74]	238.52 ± 32.02 [207.35 to 257.54]	515.41 ± 27.26 [467.97 to 556.39]	159.55 ± 8.45 [144.18 to 177.11]	0.32 ± 0.03 [0.28 to 0.43]	1.87 ± 0.17 [1.61 to 2.18]	2.06 ± 0.21 [1.67 to 2.52]	3.66 ± 0.40 [3.06 to 4.45]
60	5.89 ± 1.92 [3.12 to 8.96]	233.58 ± 31.47 [195.80 to 293.14]	533.96 ± 31.11 [485.27 to 575.14]	156.97 ± 10.24 [139.15 to 179.38]	0.30 ± 0.03 [0.27 to 0.38]	1.73 ± 0.16 [1.43 to 1.99]	2.08 ± 0.25 [1.74 to 2.57]	3.69 ± 0.36 [2.92 to 4.22]
70	5.76 ± 1.66 [3.35 to 8.06]	227.33 ± 32.51 [188.17 to 320.53]	556.09 ± 35.75 [491.98 to 618.24]	153.98 ± 10.97 [130.72 to 169.10]	0.29 ± 0.02 [0.26 to 0.37]	1.61 ± 0.17 [1.26 to 1.87]	2.07 ± 0.23 [1.80 to 2.53]	3.69 ± 0.35 [3.20 to 4.43]
80	5.56 ± 1.67 [3.14 to 8.46]	224.13 ± 38.58 [185.73 to 356.91]	579.51 ± 52.43 [508.79 to 744.17]	150.27 ± 11.62 [131.20 to 164.64]	0.28 ± 0.04 [0.24 to 0.36]	1.45 ± 0.22 [1.20 to 1.81]	2.06 ± 0.17 [1.82 to 2.35]	3.75 ± 0.34 [3.27 to 4.37]

Abbreviations: BW, body weight; BWS, body weight support; Div. Exponent, maximum divergence exponent; F_max_, body‐weight‐normalized maximum plantar force; nGCT, normalized ground contact time; PTA, peak tibial acceleration.

A correlation matrix for all measured parameters can be found in the appendix (Figure A1).

## 4 Discussion

The present study aimed to investigate biomechanical changes with varying BWS when running on an LBPPT and the differences in male and female competitive runners. The results showed that all investigated parameters were significantly affected by BWS with differences in effect sizes. While there were overall differences between male and female runners in some parameters, none revealed an interaction effect of BWS and sex.

### 4.1 Effects of BW Unloading on Running Biomechanics

#### 4.1.1 Step Rate and Normalized Ground Contact Time Are Reduced Through Unloading

Our data showed a significant decrease in step rate of 2.4 steps•min^−1^ (approx. 1.42%) with each 10% increment of BWS (*b*_BWS_ = −0.24 ± 0.01 steps•min^−1^/%BWS, *p*_BWS_ < 0.001) resulting in an 11.4% decrease from 0%BWS to 80%BWS. This is consistent with previous results [10, 11, 12]. Each 10% increment of BWS was reported with a 1.5% to 3.5% decrease in step rate per 10% increase in BWS at a self‐selected pace [12] or 2% per 10%BWS increment at a pace approximated to 75% of maximum heart rate [9]. Concordantly, Barnes and Janecke [33] found a reduction in step rate of 3.8% and 4.2% from 0%BWS to 30%BWS at 12.9 km/h for male and female runners, respectively.

Higher step rates have been linked to a reduced risk of chronic knee injuries by reducing joint loading through increased pre‐ or in‐motion muscle activation [45]. By implication, the decrease in step rate may lead to an unintentionally higher load and changed neuromuscular control, which could result in collateral injuries. Therapists may monitor step rate during rehabilitation on the LBPPT and could use a metronome if a specific step rate is required, as Stockland et al. [12] have found runners can recreate a higher step rate under BWS when instructed.

The change in swing time and stance time is partially in accordance with other results. Overall, studies agree that swing time increases with increasing BWS [10, 15, 33, 46]. Ueberschär et al. [46] found a large decrease from 0%BWS to 20%BWS and 0%BWS to 40%BWS of −2.7% and − 6.5%, respectively, for swing time, while for stance time, they observed no significant change. The decline of nGCT due to increased swing time was also implicated by Neal et al. [10]. Their measurements showed an increase in stride duration with a decrease in step rate.

For stance time, no clear picture is given since there was either no change detected [47] or it was considered minor [15] in contrast to the significant decrease found by Neal et al. [10] and Barnes and Janecke [33]. Grabowski and Kram [17] found an increase in stance time, but their study used a harness system instead of an LBPPT for BWS.

The decrease in step rate seemingly results from the increase in stride length as implied by the increased swing time in this study and previous results [11, 33, 47]. Since pace equals step rate times step length, and pace is kept constant, the unloading leads to a decrease in step rate through increasing step length by the athlete. Figure 3 illustrates the effect of BWS on the spatiotemporal parameters and how the reduction in nGCT primarily stems from an increase in swing time, rather than a reduction in stance time.

#### 4.1.2 Maximum Plantar Force Is Reduced

Our initial hypothesis of decreased maximum plantar force with increasing BWS is supported by the mixed model analysis showing a 0.01 BW (0.44%) decrease for each %BWS (*b*_BWS_ = −0.010, *p*_BWS_ < 0.001). Smoliga et al. [36] found a 0.015 BW decrease per unit of BWS for maximum in‐shoe plantar force at a comparable pace. Further studies investigating changes in peak pressure [10, 20] or vertical ground reaction force (GRF) using force plates [18] support these findings. Users and therapists should be aware that the nomenclature of the LBPPT can be misleading, such that the BW setting does not represent a percentage change in maximum forces. In our study, the maximum force at the highest support level of 80%BWS (LBPPT nomenclature: 20%BW) was approximately 64% of maximum force at 0%BWS (100%BW). The discrepancy could in part result from the general error in unweighting, as described by McNeill et al. [48]. Another explanation could be the observed reduction in stance time. The vertical impulse is the integral of the vertical GRF over the stance time (well aware that the insoles measure the normal force, not the vertical GRF). If the stance time is reduced, higher forces relative to the level of BWS are required. Since rehabilitation protocols often include a maximum weight for partial load (i.e., 20 kg partial load for 2–6 weeks after fractures of the leg) the settings on the treadmill do not provide an analog reference.

#### 4.1.3 Peak Tibial Acceleration Is Reduced With Increased Unloading

We found a significant reduction in PTA with increased unloading (*b* = −0.03, 95% CI = [−0.04 to −0.03], *p* < 0.001) which reflects a reduction of approximately 2.4 g or 30.0% from 0%BWS to 80%BWS. Previous studies, in contrast, found no significant differences in PTA when changing from no external BWS to 20% and 40% unloading in a comparable treadmill [9, 46]. A possible explanation for the different findings is the placement of the accelerometers. Both studies attached the sensors more to the distal tibia, while here it was fixated more proximally (Figure 1C). Sara et al. [49] showed that a proximal shift of the sensor led to overall lower tibial acceleration compared to a position that is more comparable to that of the other studies. Their findings only demonstrate a constant offset during overground running (no unloading). However, it is possible that the exact sensor location also affects the acceleration measured at different levels of unloading.

Tibial acceleration measured with wearable sensors is frequently used as a proxy for tibial bone loading and was strongly correlated with vertical impact loading rates [50]. Loading rates, in turn, were greater in female runners who sustained bone stress injuries in a prospective cohort study [51]. Our results indicate that reducing the weight force during running using an LBPPT is an effective measure to reduce tibial stress and strain. Of note, recent findings showed a weak correlation between PTA and maximum tibial bone compression force [52]. The authors suggested careful consideration of PTA as an indicator of bone stress and strain. However, they only reported maximum tibial bone load and not compressive loading rates, which are known to affect bone material properties [53]. Regardless of the method used to assess bone stress, partial weight‐bearing is a standard of care for rehabilitating lower extremity bone stress injuries in runners [54]. The findings of this study, therefore, support the use of LBPPT during the early stages of rehabilitation when overground running is not yet permitted. Nevertheless, further research is needed to explore best practice strategies for implementing LBPPT in return‐to‐sports protocols following bone stress injuries.

#### 4.1.4 Running Stability Is Only Marginally Affected

Running stability, defined as the ability to withstand small perturbations during bipedal locomotion, was anticipated to improve with increased BWS. Unloading reduces the required work to support one's own BW, which was expected to yield a more stable gait. In our study, we observed that at the foot, running stability indeed increased with higher BWS levels (*b* = −0.001, 95% CI: [−0.002, 0.000], *p* = 0.017). Conversely, at the tibia, we found a slight but significant decrease in running stability with increased BWS (*b* = 0.001, 95% CI: [0.000, 0.002], *p* = 0.009). Both effects, while statistically significant, were minimal. Santuz et al. [22] investigated treadmill running on an even and uneven surface. They found that running stability was significantly reduced in the more challenging uneven conditions. Another study showed reduced running stability when transitioning from shod to barefoot treadmill running [23]. To our knowledge, no prior studies have examined running stability under a *less challenging* condition compared to regular running. It is possible that running stability was already at an optimal or maximal level under the 0%BWS condition in our study, so additional unloading did not lead to substantial improvements.

### 4.2 No Sex‐Specific Responses to Running With Unloading

None of our analyses showed a significant interaction effect of BWS and sex. This indicates that there are no differences in the response to BWS in running biomechanics between male and female runners on the LBPPT. Similar results were reported by Banes and Janecke [33] who investigated male (*n* = 7) and female (*n* = 8) distance runners. They found similar responses in step rate, contact time, and swing time between both sexes. Other studies examining LBPPT either did not include females [10, 11, 14, 18, 48] or did not perform sex‐specific data analyses [12, 25]. The biomechanical sex differences in distance running are well documented [55] and some could be observed in our results. As injury rates may differ between female and male runners [2], it is therefore important to understand potential sex‐specific responses to interventions like the LBPPT. Our results show that both sexes respond similarly to BW unloading, indicating that sex‐specific LBPPT training programs do not necessarily have to be adapted.

### 4.3 Outlook and Practical Implications

The results of this study have important clinical implications for users and therapists. Even though the antigravity treadmill is gaining popularity and thus availability, implementation of the LBPPT should be done with consideration to the individual's needs and goals. While the general reduction of musculoskeletal load seems to be given for various force parameters, accompanying changes, for example, in step rate and nGCT could ultimately influence neuromuscular patterns and lead to an unintentional change of gait pattern. The body needs to adapt gradually to any (reoccurring) changes in stimuli. Additionally, different tissues (i.e., muscles and tendons) require different durations and stimuli for adaptation [56]. The biomechanical changes found in this study could be overstraining in the sense of novel exposure for specific regions of the foot and leg. Studies examining short‐ and long‐term effects on additional running mechanics and following an LBPPT training back to overground running are needed to clarify those risks. Since step rate can be successfully modulated voluntarily as shown by Stockland et al. [12], the interaction between force and other biomechanics should be evaluated. Some characteristics that might need to be maintained for competitive runners, that is, step rate, could influence the overall load. For early rehabilitation, the use of measuring insoles could be generally advised. The specificity of the training could be monitored as they offer a transportable and real‐time measurement of pressure values as well as a variety of posttraining analyses. Prevention and rehabilitation on the LBPPT should be based on the individual's running style and risk profile even though our results showed that female and male runners respond similarly to the unloading. Overall injury rates might not differ significantly between sexes, but female runners tend to sustain more bone stress injuries than their male counterparts [2]. Nevertheless, more sex‐sensitive data are needed for a conclusive evaluation.

### 4.4 Strengths and Limitations

There are some limitations to this study. Variability in foot strike patterns and running shoes was not specifically examined since peak plantar force was the only parameter directly affected. Additionally, the sample consisted of healthy participants only. Studies on injured runners are needed to investigate possible differences due to injuries. The detection of gait events was also challenging since some datasets controversially showed no complete unloading of the insoles at higher support settings. This may result from participants gripping their toes or tensing their feet. The threshold was hence computed at 50 N instead of the commonly used 20 N to improve the robust detection of gait events. For some datasets, this might still have resulted in inaccurate gait event estimation. To minimize this effect, values were averaged over 30 s with enough spacing to interval borders to also reduce possible errors from the visual increment detection. In general, insoles can only provide the normal force component and thus do not include eventual shear forces that may be relevant for injuries. Thus, we cannot conclude on the additional effects of unloading on horizontal ground reaction forces during the stance phase. In our study, we only used a single constant speed for all participants. The standardized speed of 12 km/h was chosen to achieve comparison, especially of the spatiotemporal parameters, to avoid fatigue during the running task and to simulate a clinically relevant speed during rehabilitation approaches. This, however, limits the generalizability of our findings to other running speeds.

## 5 Perspectives

Rehabilitation after running‐related injuries requires careful management of incremental load progression. LBPPTs enable precise control over external loads. Understanding how this unloading affects running biomechanics is crucial for targeted interventions. Here we demonstrated that the level of BWS when running on such a device affects several biomechanical parameters. The external load on the lower limbs was generally reduced through increased BWS as shown by reduced peak tibial acceleration and maximum plantar force. Changes in spatiotemporal parameters revealed a shorter ground contact time, along with longer steps. These findings closely align with previous studies [10, 36]. Additionally, our results provide new insights into nonlinear and sex‐specific responses to unloading. Although running stability was significantly affected, the small effect size raises questions about its clinical relevance. Female and male runners exhibited similar responses to unloading, but there were overall differences in maximum plantar force and step rate. Our results provide a detailed description of changes in running biomechanics in a population that is at risk of sustaining running‐related injuries. This can help inform practitioners and therapists to choose optimal training strategies when a LBPPT is available.

## Author Contributions

**Dominik Fohrmann:** writing – original draft, methodology, investigation, formal analysis, data curation, conceptualization. **Isabelle Winter:** writing – original draft, investigation, formal analysis. **Alexander Simon:** writing – review and editing, methodology, Investigation. **Dimitris Dalos:** writing – review and editing. **Thomas Gronwald:** writing – review and editing. **Tim Hoenig:** writing – review and editing, conceptualization. **Tim Rolvien:** writing – review and editing, methodology, conceptualization. **Karsten Hollander:** writing – review and editing, supervision, project administration, methodology, conceptualization.

## Conflicts of Interest

The authors declare no conflicts of interest.

## Supporting information


**Figure A1.** Correlation matrix of the investigated biomechanical parameters. Values in the fields are Pearson’s product–moment correlation coefficient and *p* value on top and bottom of each cell, respectively. Cell shading intensity reflects the correlation coefficient with darker fill colors indicating higher absolute values.

## Data Availability

The data that support the findings of this study are available on request from the corresponding author. The data are not publicly available due to privacy or ethical restrictions.
