# Supplementary material for: Biomechanical Changes During Running on a Lower Body Positive Pressure Treadmill in Competitive Runners
Source: Scand J Med Sci Sports. 2025 May 11;35(5):e70063. doi: 10.1111/sms.70063 (PMC12066903; doi:10.1111/sms.70063)
Supplement: Supplementary file 1 — Figure A1. Correlation matrix of the investigated biomechanical parameters. Values in the fields are Pearson’s product–moment correlation coefficient and p value on top and bottom of each cell, respectively. Cell shading intensity reflects the correlation coefficient with darker fill colors indicating higher absolute values. [file SMS-35-e70063-s001.docx]

**Supplementary**


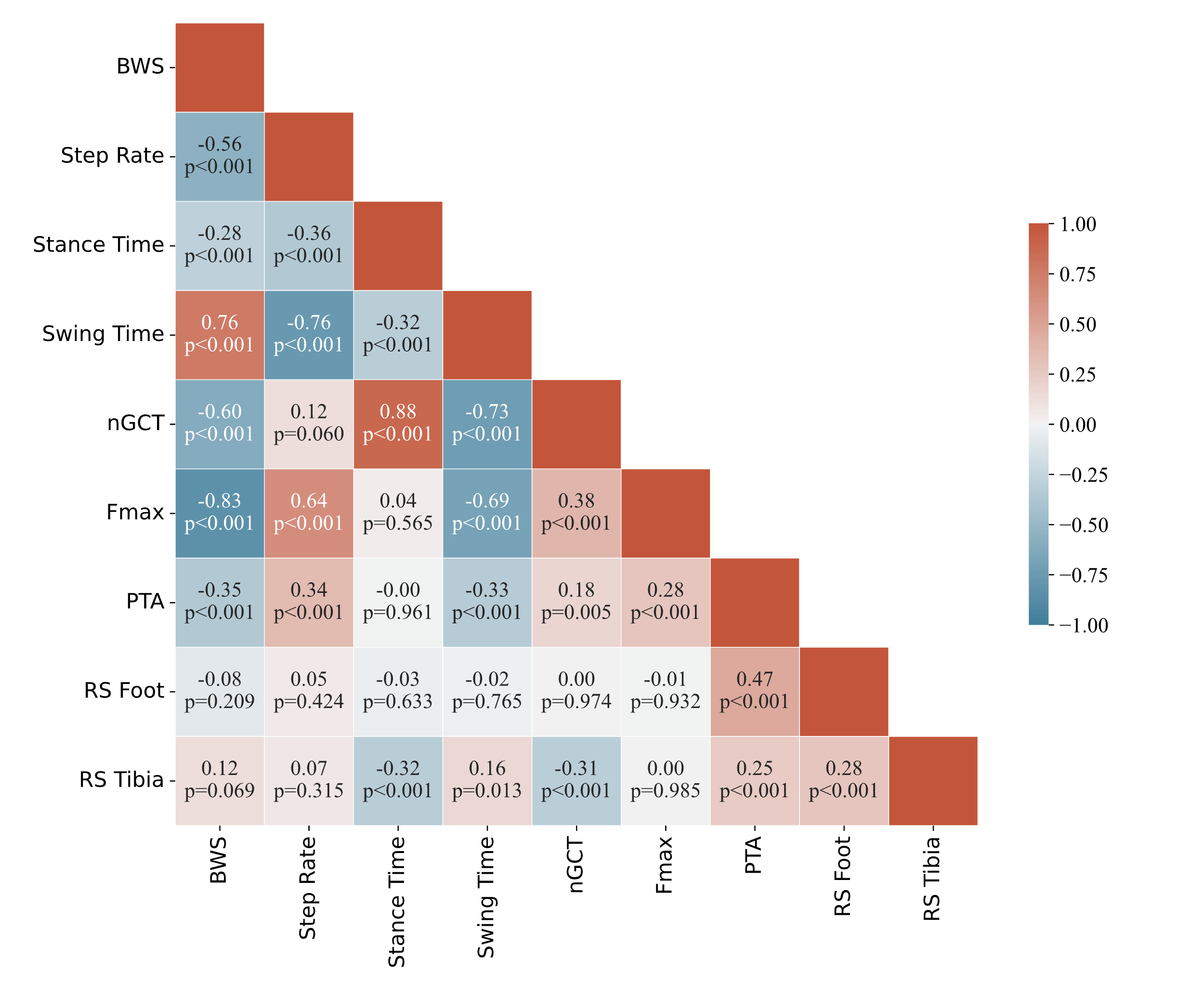


**Fig. A1:** Correlation matrix of the investigated biomechanical parameters. Values in the fields are Pearson’s product-moment correlation coefficient and p-value on top and bottom of each cell respectively. Cell shading intensity reflects the correlation coefficient with darker fill colors indicating higher absolute values.
